# Identifying barriers and enablers to cardiac rehabilitation participation and completion unique to South Asian individuals: a qualitative systematic review

**DOI:** 10.1093/eurjcn/zvaf044

**Published:** 2025-03-21

**Authors:** Joanne McAllister, Mary Harrison, Claire A Lawson, Sally J Singh

**Affiliations:** Respiratory Department, CERS 2 Office, Glenfield Hospital, University of Leicester, Leicester, Leicestershire LE3 9QP, UK; Centre for Exercise and Rehabilitation Science (CERS), Leicester Biomedical Research Centre (Respiratory), Glenfield Hospital, Groby Road, Leicester LE3 9QP, UK; Department of Cardiovascular Sciences, University of Leicester, Leicester LE3 9QP, UK; Leicester Biomedical Research Centre (Cardiovascular), Glenfield Hospital, Groby Road, Leicester LE3 9QP, UK; Department of Cardiovascular Sciences, University of Leicester, Leicester LE3 9QP, UK; Leicester Biomedical Research Centre (Cardiovascular), Glenfield Hospital, Groby Road, Leicester LE3 9QP, UK; Respiratory Department, CERS 2 Office, Glenfield Hospital, University of Leicester, Leicester, Leicestershire LE3 9QP, UK; Centre for Exercise and Rehabilitation Science (CERS), Leicester Biomedical Research Centre (Respiratory), Glenfield Hospital, Groby Road, Leicester LE3 9QP, UK

**Keywords:** Barriers, Cardiac rehabilitation, Enablers, South Asian

## Abstract

**Aims:**

South Asian individuals living outside the Indian subcontinent are under-represented in cardiac rehabilitation (CR), despite facing higher rates of cardiac-related mortality and hospitalizations compared with White ethnic groups. Understanding how cultural differences affect CR participation and completion after referral requires the examination of barriers and enablers specific to South Asian populations. This review aims to summarize evidence on barriers and enablers to CR for South Asian minorities outside the Indian subcontinent.

**Methods and results:**

A systematic review of six databases (MEDLINE, CINAHL, APA PsycINFO, Cochrane, Scopus, and Web of Science) was conducted in January 2024 (updated November 2024), with no language or date limitations. Studies from countries outside the Indian subcontinent were included. Barriers and enablers were identified, followed by thematic analysis. Thirteen studies (*n* = 384 South Asian participants) from the UK (*n* = 10) and Canada (*n* = 3) were included. Five key themes were generated: (i) communication and knowledge, (ii) motivation to attend CR or follow medical advice, (iii) religion, (iv) programme delivery, and (v) practical considerations. Key factors influencing CR participation included language barriers, family support, fatalistic beliefs, and motivation. Each theme encapsulates barriers and enablers and shapes participation at both service and patient level. While some barriers were common across different ethnic groups, they had stronger impact on South Asians due to distinct social determinants of health.

**Conclusion:**

Barriers and enablers are closely linked to cultural and societal norms. Recognizing these factors enables CR services to better address the needs of underserved populations, ensuring equitable healthcare provision and support.

**Registration:**

PROSPERO CRD42023482611

## Graphical Abstract

**Graphical Abstract:**
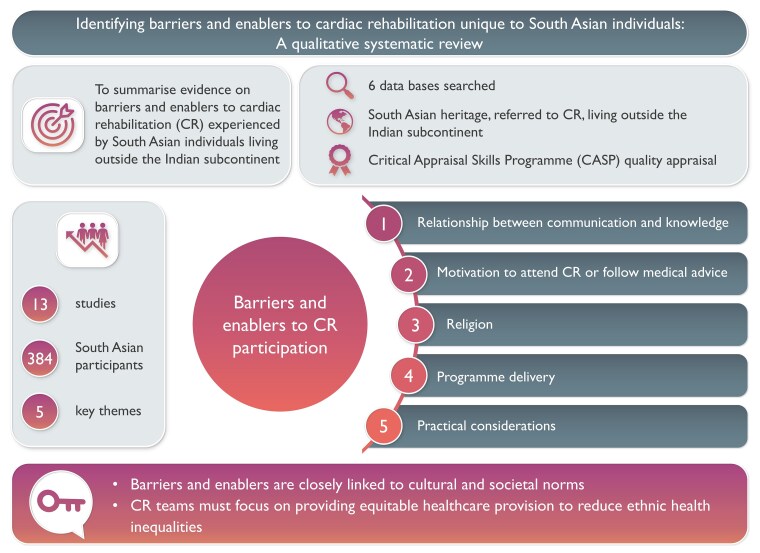

Novelty

- This study specifically examines South Asian cardiac rehabilitation (CR) service users living in countries outside the Indian subcontinent. Their unique cultural and societal factors influencing CR participation after referral have not been comprehensively reviewed due to data being often aggregated as ‘ethnic minorities’.
- South Asian CR service users experience specific barriers such as language issues, cultural and religious influences, extrinsic motivational drivers and family dynamics that affect their participation in CR, with some factors having a greater impact compared with White ethnic groups due to their interaction with social determinants of health. This nuanced understanding is crucial for designing tailored interventions.
- By focussing on the intersection of cultural norms and healthcare engagement, the study provides actionable insights for the development of culturally sensitive strategies to increase CR participation after referral, enabling the delivery of equitable healthcare.

## Introduction

Cardiovascular disease (CVD) remains the leading cause of global mortality, posing significant health challenges, particularly among ethnic minorities.^1^ South Asians, encompassing individuals from a diverse group originating from India, Pakistan, Bangladesh, Sri Lanka, Bhutan, Maldives, Nepal, and Afghanistan (Indian subcontinent),^2^ experience higher rates of CVD than other ethnic groups, including White European and Black African or Caribbean populations.^3^ This heightened risk affects South Asians both within and outside the Indian subcontinent.^4^ Cardiovascular disease impact is often more severe for South Asians livings in Western countries, where prevalence rates can be three to four times higher than in majority populations, due to higher incidence of established risk factors such as hypercholesterolaemia, excess abdominal obesity, and insulin resistance.^5,6^ Both genetic factors, such as smaller coronary vessel size, and modifiable risk factors, such as low physical activity and high fat diets, contribute to South Asians high CVD risk.^7,8^

The complex interplay of genetic, environmental, and lifestyle factors calls for targeted interventions like cardiac rehabilitation (CR) to mitigate modifiable risks and improve outcomes. Cardiac rehabilitation is a structured programme providing physical, psychological, and educational support and has been shown to reduce cardiovascular mortality and improves quality of life.^9^ However, CR is under-utilized with only 50% of referred patients attending an exercise-based programme in the UK,^10^ and with global uptake rates between 20 and 50%.^11^ Nanayakkara *et al*.^12^ compared outcomes from 811 South Asian and 5406 White Canadian individuals and found greater attrition rates for exercise and education components of CR, and lower functional capacity improvements following completion among South Asian participants. When South Asian individuals do attend CR in high income countries with good CR provision, they experience improved mortality and morbidity, though to a lesser extent than the majority population.^13^ Reasons for reduced improvement are largely unknown due to inconsistent ethnicity recording. A Cochrane review^9^ appraising evidence on exercise-based CR included 85 studies, of which 69 did not record ethnicity. The studies may have had significant ethnic diversity but the lack of recording creates uncertainty. Research suggests culturally tailored adaptations are needed so all ethnic populations can equitably benefit.^14,15^ Following these adaptations, effectiveness needs to be re-evaluated to assess CR effectiveness for South Asian participants.

Improvements in the CR referral process are needed. However, given the low percentage of referred patients who actually participate, it is equally critical to explore the barriers and enablers affecting participation and completion of core CR components, such as exercise and education sessions. Collaboration between CR service users and clinicians becomes essential to overcome barriers, ensuring better engagement among those already referred. Key guidelines and standards ask services to deliver adaptable programmes, tailored to individual needs of underserved populations.^16,17^ However, a lack of research relating to ethnic specific barriers prevents guidelines from recommending effective service adaptations. Often, multiple ethnicities are aggregated together under the collective term of ‘ethnic minorities’, making it difficult to consider cultural variations seen between ethnic populations. Whilst disaggregating data into smaller groups, such as ‘South Asian’, offers a more nuanced understanding, it still falls short of capturing the intricate intra-ethnic differences within these smaller categories. Nevertheless, this approach represents an improvement over treating all ethnic minorities as a single, homogeneous group. The South Asian community encompasses multiple distinct ethnic, linguistic, religious, and cultural groups, each with unique health beliefs, socioeconomic statuses (SESs), and healthcare experiences. Further disaggregation of data at a sub-ethnic level would be optimal, however not currently possible due to how ethnicity is currently recorded and reported in the majority of studies addressing CR participating.

Many barriers identified for minority populations overlap with those faced by White European populations,^18^ however, not all ethnicities will experience these barriers equally due to differences in social determinants of health. For example, low health literacy serves as a barrier to CR participation by limiting service users understanding of its benefits and their ability to navigate healthcare services.^19^ It is also associated with higher hospital admission rates, increased anxiety, and reduced quality of life,^20^ highlighting a population in great need of CR, yet less likely to engage. Whilst low health literacy is present across all populations, it is disproportionately observed among South Asian individuals in the UK.^21^ It is also more prevalent among those with lower SES and multiple long-term conditions, which are more widespread in South Asians living in the UK.^21,22^ Although barriers to CR participation exist across ethnic groups, the influence of social determinants of health shapes their impact, resulting in disparities in CR engagement. Furthermore, some barriers to participation after referral are specific to South Asian populations, as they are often integrated with religion, and societal and cultural norms. While barriers are widely studied, enablers such as family involvement, clinician encouragement, and community-based CR locations, must be emphasized to improve uptake and completion.^23,24^ These factors are not well understood, placing CR programmes at risk of providing care that is not culturally congruent, and that does not meet the unique needs of South Asian communities, thereby widening ethnic health inequalities.

A recent systematic review^23^ also explored South Asian CR participation, however our comprehensive review identified four additional studies^24–27^ which doubled the sample size to a total of 384 participants, strengthening the robustness of our findings. Notably, one paper^24^ was a large, multi-centred study focussing on facilitators to CR, delineated by ethnicity. Similarly, a review by Carew Tofani *et al*.^15^ addressed experiences from multiple ethnic minority populations from only seven studies, with some populations represented by a very small numbers of participants. Additional data included in the present review, alongside a specific focus on South Asian individuals, allowed our thematic analysis to go beyond the scope of the previous reviews by generating global themes that consider barriers, enablers, and/or associated influences related to societal and cultural norms of South Asian populations during CR engagement.

Addressing the under-representation of South Asian individuals in CR programmes is essential for improving cardiovascular outcomes within this high-risk group. Clinicians should consider both barriers and enablers to participation when considering programme adaptations. This systematic review aims to examine prominent and unique barriers and enablers associated with CR participation after referral among South Asian ethnic minorities.

## Methods

### Data sources and search strategy

Six databases were searched (January 2024, searches re-run in November 2024) that focused on barriers and/or enablers to CR experienced by South Asian CR service users. Search terms for ‘South Asian’ and ‘cardiac rehabilitation’ were developed and validated in MEDLINE. Terms were then refined and applied to the following databases: MEDLINE (OVID including PubMed), CINAHL, APA PsycINFO, CENTRAL, Scopus, and Web of Science (see Supplementary material online, *Table S1*). Reference lists and relevant reviews were hand searched. No date restrictions were applied, as the recording of ethnicity within research has been implemented over a widespread timeline.

### Selection criteria

Primary research that explored barriers and/or enablers to CR exercise or education programmes was included. Studies in all languages were included providing abstracts were written in English. Studies included adult (≥18 years old) participants with South Asian heritage currently residing outside of the Indian subcontinent. Studies could also include other ethnicities, however only outcomes for South Asian participants were extracted, and therefore these outcomes had to be discernible from other ethnic groups. All cardiac events were considered for inclusion, provided they resulted in a referral to CR. Exclusion criteria were: case studies, review articles, grey literature and pilot studies, research on generic primary prevention exercise programmes, paediatric or animal studies, research conducted in the Indian subcontinent and studies which did not include South Asian ethnic minorities.

### Study screening and data extraction

Titles and abstracts were screened independently by two authors (J.M. and M.H.), and the remaining full text articles were independently reviewed by the same two authors. Conflicts were resolved through consultation with a third reviewer (C.A.L.), a senior researcher who holds extensive experience in conducting systematic reviews. Study screening was conducted in Covidence. Data extraction was guided by the *Cochrane Handbook for Systematic Reviews of Interventions* (2024)^28^ and included: year of publication, author, study design, setting, number of participants, study aim, data collection, sampling method, participants ethnicity, and key barriers and enablers to CR uptake, adherence, and/or completion.

### Quality appraisal

As only qualitative studies were found following the screening process, study quality was independently appraised using the Critical Appraisal Skills Programme (CASP) for qualitative studies.^29^ Critical Appraisal Skills Programme is a commonly used, validated appraisal tool for qualitative research. It considers various methodologies such as interviews and focus groups and its use is supported by Cochrane and World Health Organization guideline processes.^30^ Studies were scored out of 10 and rated as good (8–10), fair (5–7), or poor (1–4)^14^ (*Table 1*).

**Table 1:** Summary of Critical Appraisal Skills Programme quality assessment

	Was there a clear statement of the aims of the research?	Is a qualitative methodology appropriate?	Was the research design appropriate to address the aims of the research?	Was the recruitment strategy appropriate to the aims of the research?	Was the data collected in a way that addressed the research issue?	Has the relationship between researcher and participants been adequately considered?	Have ethical issues been taken into consideration?	Was the data analysis sufficiently rigorous?	Is there a clear statement of findings?	How valuable is the research?	CASP rating (/10)
Astin, Atkin and Darr (2007)	Y	Y	Y	Y	Can’t Tell	Can’t Tell	Can’t Tell	Y	Y	A valuable exploration of participants experiences, with clear inferences of the impact on clinical practice but needed more methodological detail	7/10 Fair
Banerjee *et al*. (2010)	Y	Y	Y	Y	Y	N	Y	Can’t Tell	Y	Adds value to the insights of participants experience with good recommendations for clinical practice	8/10 Good
Chauhan *et al*. (2010)	Y	Y	Y	Can’t Tell	Y	Can’t Tell	Y	Y	Y	A valuable research which provided insight into the topic, using robust qualitative methods	8/10 Good
Darr, Astin and Atkin (2008)	Y	Y	Y	Can’t Tell	Y	N	Can’t Tell	Y	Y	A valuable study contributing to the pool of research however lacked detail on researcher positionality and informed consent	7/10 fair
Dilla *et al*. (2020)	Y	Y	Y	Can’t Tell	Y	Y	Y	Y	Y	A relevant study regarding the current landscape of CR and health inequalities but lacked exploration of the findings impacts on to clinical practice	9/10 Good
Galdas and Kang (2010)	Y	Y	Y	Y	Can’t Tell	N	Y	Can’t Tell	Y	Research limited to a very small population and the use of grounded theory needed further exploration	7/10 Fair
Grewal *et al*. (2010)	Can’t Tell	Y	Can’t Tell	N	Can’t Tell	N	Y	Can’t Tell	Can’t Tell	Poor methodology and lack of detail on the recruitment and interview processes limits the researches usability	4/10 Poor
Jolly *et al*. (2007)	Y	Y	Y	Can’t Tell	Can’t Tell	N	Y	Can’t Tell	Y	The research was novel at the time of completion and addressed the needs of the local South Asian population, however, lacked detail on methodology and analysis	5/10 Fair
Lotto *et al*. (2022)	Can’t Tell	Y	Y	N	Can’t Tell	N	Y	N	Y	The value of the research was limited by under-developed themes and a very small sample size	4/10 Poor
Sharif (2020)	Y	Y	Y	Can’t Tell	Y	Can’t Tell	Y	Can’t Tell	Can’t Tell	A valuable addition, however, findings would have been better condensed and summarized into main themes and further use of findings explored	6/10 Fair
Tod *et al*. (2001)	Can’t Tell	Y	N	Can’t Tell	N	N	N	Can’t Tell	Y	Possibly due to age, this research lacked value due to small sample with little exploration of the topic area and impacts of the results	3/10 Poor
Visram *et al*. (2008)	Y	Y	Y	Can’t Tell	Can’t Tell	N	Y	Can’t Tell	Y	Valuable research which provides insight into barriers and enablers to CR participation	6/10 Fair
Webster, Thompson and Mayou (2002)	Y	Y	Y	Can’t Tell	Y	N	Y	Can’t Tell	Can’t Tell	Valuable insight into a very specific group of South Asian participants but little recommendation on impact of the findings	5/10 Fair

### Analysis of outcomes

**Figure 1:**
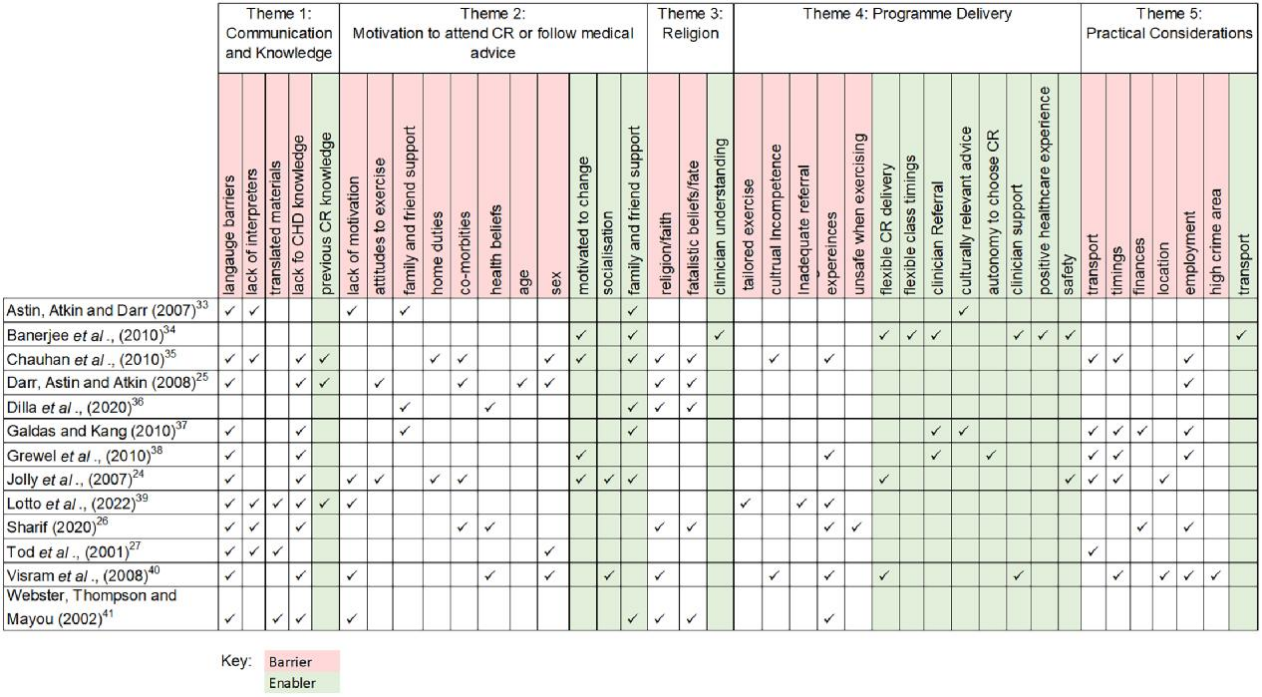
Theme matrix.

Thematic analysis, following the six stages outlined by Braun and Clarke,^31^ was used to synthesize the results. Text data from results, conclusion, and discussion sections of each study were analysed, which included quotations and author interpretations. Data from four studies were inductively coded line-by-line to search for concepts, independently by two reviewers. Initial codes were generated based on their relation to barriers and enablers. These codes were then discussed by the reviewers to check interpretations of the text and gain consensus.^32^ The remaining studies were coded using the same codes, however, new codes were generated alongside as topics arose. Multiple codes were assigned to each text section, then grouped into broader descriptive themes and finally into global themes^32^ (*Figure 1*). This iterative process was completed as a discussion between the two reviewers, with any disagreements resolved through consensus. This synthesis provides a flexible approach, allowing themes to be data driven without restrictive *a priori* hypotheses,^32^ pertinent given the sociocultural differences between participants and researchers. Analysis was carried out in Nvivo 12 which allowed enhanced visualization of codes linked directly to original transcripts. If codes existed under multiple themes, they were placed in the most prominent theme.

### Reporting and registration

The review followed the Preferred Reporting Items for Systematic Reviews and Meta-Analyses (PRISMA) guidelines and the Enhancing Transparency in Reporting the Synthesis of Qualitative Research (ENTREQ) statement. The protocol was registered on PROSPERO (CRD42023482611), review protocol available.

### Patient and public involvement

Multiple public engagements and informal discussions with South Asian health service users, and members of the public, informed the question development and interpretation of findings.

## Results

### Study characteristics

**Figure 2:**
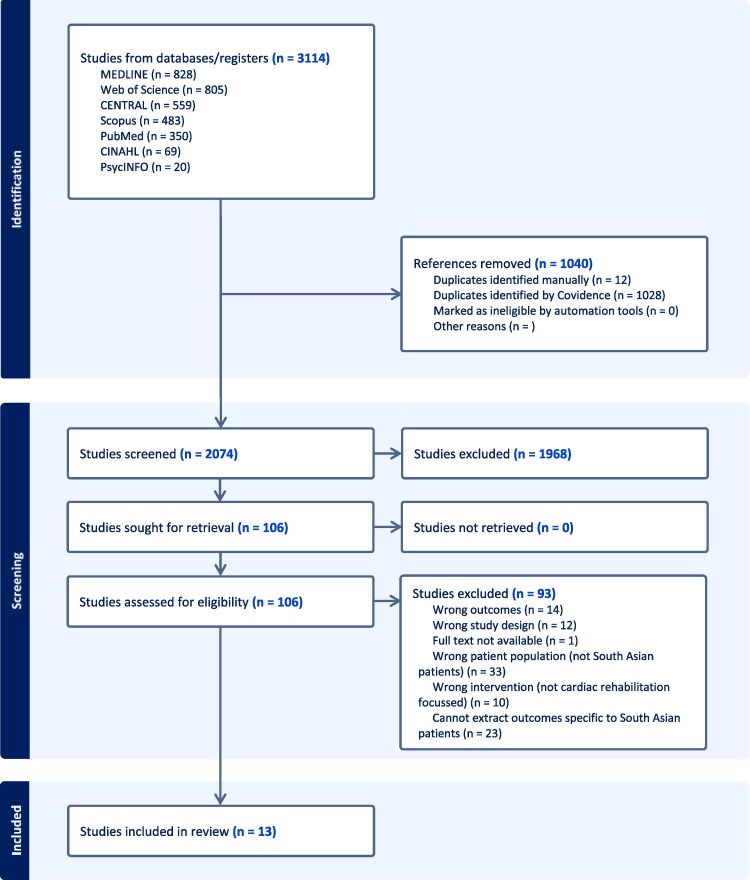
Study selection process.

**Figure 3:**
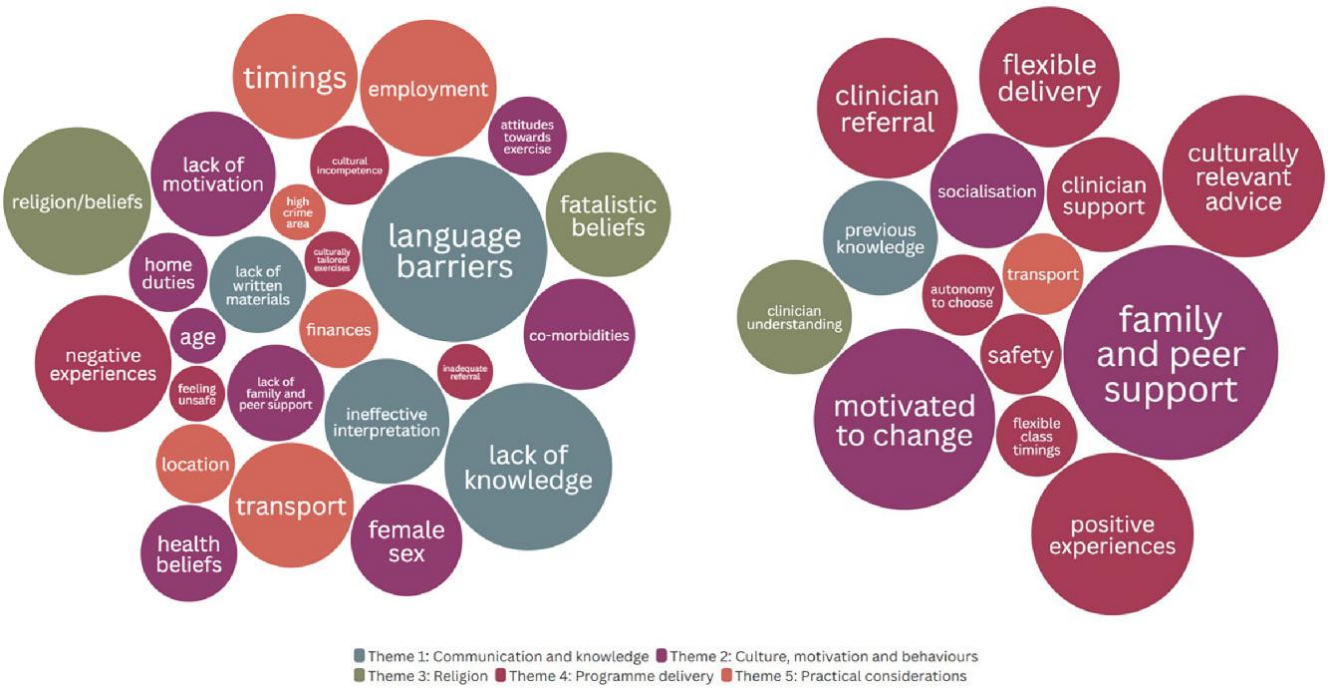
Barriers and enablers to cardiac rehabilitation reported by South Asian service users (size of bubble indicates frequency of reporting). Figures were created by Flourish visualization software (https://flourish.studio/).

**Figure 4:**
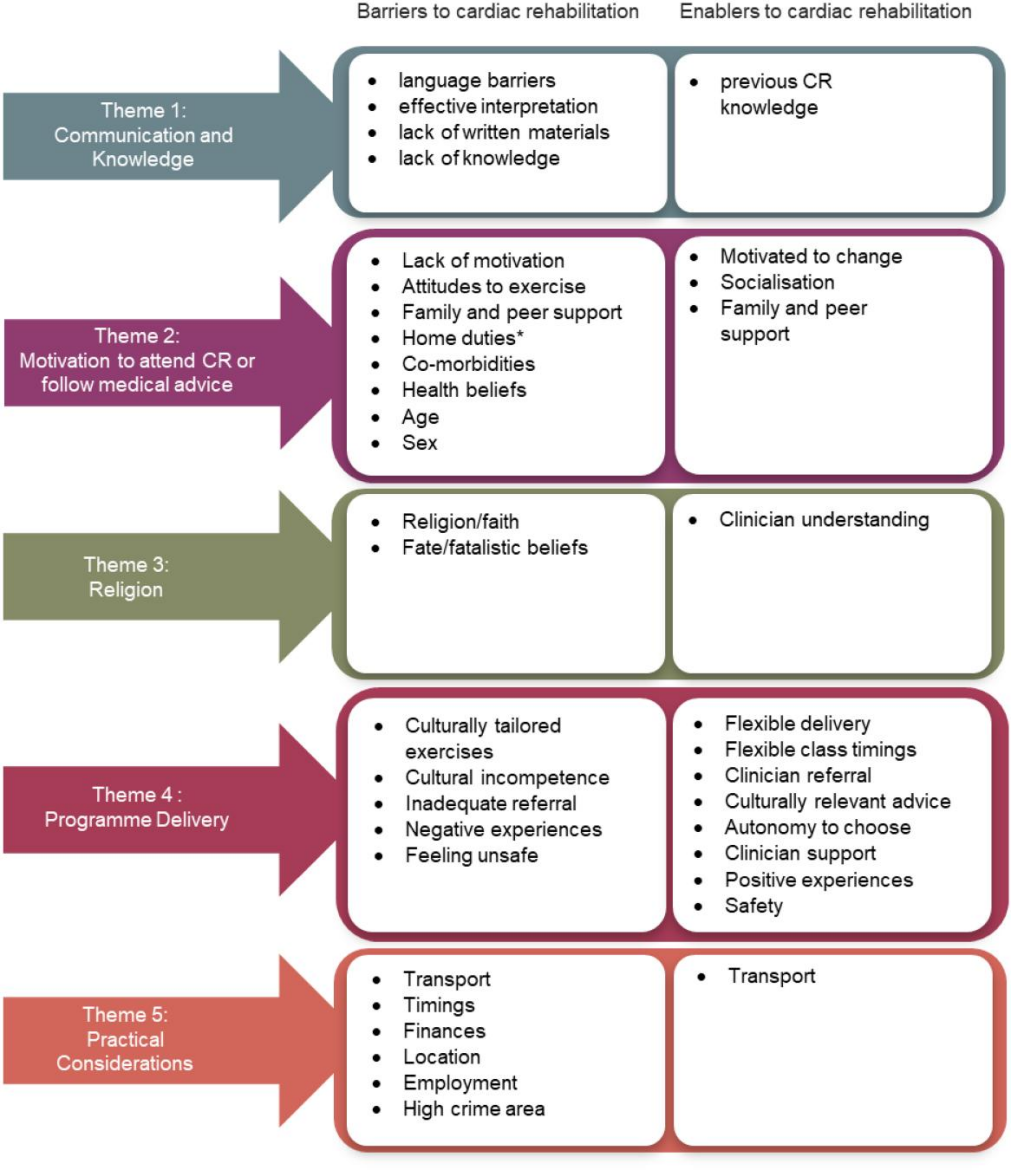
Themes with associated barriers and enablers to cardiac rehabilitation.

Of the 3114 studies screened, 2074 remained after de-duplication and 106 remained after abstract screening. Following full text review, 13 studies were identified for inclusion in the review^24–27,33–41^ (*Figure 2*). These studies included 384 South Asian participants from India, Pakistan, Bangladesh, and Sri Lanka. Study characteristics are presented in *Table 2*. All studies employed a qualitative approach, with 11 studies^24–27,33–41^ using semi-structured interviews, one study using a focus group^40^ and one study^27^ using a descriptive audit questionnaire with narrative synthesis. Studies were from the UK (*n* = 10) and Canada (*n* = 3). Five key themes were generated: (i) communication and knowledge, (ii) culture, motivation and behaviours, (iii) religion, (iv) programme delivery, and (v) practical considerations {*Figure 3* [Figures were created with the data visualization software Flourish (https://flourish.studio/)]; *Figure 4*}. A theme matrix is presented in *Figure 1*. Each theme represents an overall topic which encapsulates elements of barriers and/or enablers, or wider influencing factors that can either promote or hinder participation, operating at the patient and healthcare system levels.

**Table 2:** Study summary table with quality appraisal scores

Author	Year/location	Sample size	Age	Sex	Ethnicity	CVD history	Design	Key outcomes	Quality appraisal
Astin *et al*.^33^	2008, UK	65 participants	62 years	36 males (55%) 29 females (45%)	Pakistani Muslim (20), Indian Sikh (12), Indian Hindu (13) and White European (20)	Unstable angina, MI or CABG	Qualitative Semi-structured interviews	*Barriers:* Language barriers Lack of family support due to work Transport *Enablers*: Family support Help with dietary changes	CASP: 7/10 Rated: Fair
Banerjee *et al*.^34^	2010, Canada	16 participants	57 years	13 males (81%) 3 females (19%)	South Asian	MI, PCI, CABG, heart failure, valve replacement	Qualitative Semi-structured interviews	Enablers: *Pre-disposing:* Family and friend support Motivation Medically supervised exercise classes Previous CR knowledge *Enabling:* Transport Referral from senior clinician (physician) Flexible timings *Reinforcing:* Positive healthcare experiences Clinician support Family and friend support	CASP: 8/10 Rated: Good
Chauhan *et al*.^35^	2010, UK	20 participants	59 years	13 males (65%) 7 females (35%)	MI, PCI, CABG, heart failure, valve replacement	Pakistani (12), Indian (6) and Bangladeshi (2)	Qualitative Semi-structured interviews	*Barriers:* Patient Level: Lack of CHD knowledge Sex Religion Language barrier Practical Considerations (time, money, transport) Negative healthcare experiences Fatalistic beliefs/religion Poor recognition of CR nurse role Social networks Provider Level: Stereotyping Lack of cultural competence	CASP: 8/10 Rated: Good
Darr *et al*.^25^	2008, UK	65 participants	63 years	36 males (55%) 30 females (45%)	Unstable angina, MI or CABG	Pakistani Muslim (20), Indian Sikh (12), Indian Hindu (13) and White European (20)	Qualitative In-depth semi-structured interviews Framework approach (coding framework)	*Barriers*: Language barriers Religion/culture Age/Sex Logistical factors Perceptions of exercise CHD knowledge	CASP: 7/10 Rated: Fair
Dilla *et al*.^36^	2020, UK	14 participants	56 years	9 males (65%) 5 females (35%)	MI	South Asian	Qualitative Constructivist grounded theory	*Barriers:* Fatalistic beliefs/religion Health Beliefs Family commitments Culture *Enablers:* Family and friend support Balance between health and family	CASP: 9/10 Rated: Good
Galdas and Kang^37^	2010, Canada	15 participants	45–80 years	10 males (67%) 5 females (33%)	MI	Punjabi Sikh	Qualitative: Thematic analysis with grounded theory	*Barriers:* Lack of knowledge on prognosis and management Fatalistic beliefs Transport Language barriers *Enablers:* Practical dietary advice Peer socialization Interaction with CR team	CASP: 7/10 Rated: Fair
Grewal *et al*.^38^	2010, Canada	16 participants	63 years	15 males (94%) 1 female (6%)	ACS	Indian (11), 2 Bangladeshi (2), Sri Lankan (1) and Ugandan (1)	Qualitative Semi-structured interviews	*Barriers:* Lack of interaction with clinicians prior to discharge Lack of CR knowledge *Enablers:* Effective referral Patient choice to attend CR Positive attitude towards CR programme	CASP: 4/10 Rated: Poor
Jolly *et al*.^24^ (qualitative aspect of study only)	2007, UK	49 participants	63 years	33 males (67%) 16 females (33%)	MI, PCI, CABG	White British/Irish (39), Indian (7), Pakistani (2), Black Caribbean (1)	Qualitative Semi-structured interviews	*Barriers:* Lack of exercise experience Family/home duties Lack of motivation Currently active Co-morbidities Location/timings *Enablers:* Supervised programme Home based is convenient Socialization Engaged in lifestyle modification Safety of hospital location	CASP: 5/10 Rated: Fair
Lotto *et al*.^39^	2022, UK	6 participants	Not reported	5 males (83%) 1 female (17%)	PCI, CABG	South Asian	Qualitative Semi-structured interviews	*Barriers:* Language barrier Lack of structured CR programme Poor referral process Lack of CR knowledge Lack of interest in CR Lack of culturally appropriate exercises	CASP: 4/10 Rated: Poor
Sharif^26^	2020, UK	18 participants	19–79 years	13 males (72%) 5 females (28%)	Heart Failure	Pakistani	Qualitative Semi-structured interviews with grounded theory	*Barriers:* Negative healthcare experiences fear about pushing themselves too far financial costs weather status employment status mobility status accessing information *Enablers:* Positive healthcare experiences	CASP: 6/10 Rated: Fair
Tod *et al*.^27^	2001, UK	76 participants	Not reported	Not reported	MI, CABG, angina, other cardiac complaints	Indian, Pakistani, Bangladeshi, Other Asian	Qualitative descriptive audit with narrative synthesis	*Barriers*: Lack of interpreter services lack of translated written materials mobility transport issues	CASP: 3/10 Rated: Poor
Visram *et al*.^40^	2008, UK	9 participants and 8 healthcare workers	Not reported	17 females (100%)	Not reported	Pakistani, Bangladeshi	Qualitative Semi-structured interviews and focus group	*Barriers:* *Individual Barriers:* Lack of CR knowledge Negative healthcare experiences Lack of motivation Exercise will cause discomfort/unsafe *Cultural Factors:* Language barrier Exercising alongside males Perceived inappropriate topics *Personal Factors:* Location Employment Clash with prayer times Safety due to high crime area *Enablers:* Motivation from healthcare professionals Qualified staff/safety Involvement of family members	CASP: 6/10 Rated: Fair
Webster *et al*.^41^	2002, UK	35 participants	65 yrs	25 males (71%) 10 females (29%)	MI	South Asian (Gujarati Hindu)	Qualitative Semi-structured interviews	*Barriers:* Lack of CR knowledge Lack of motivation Language barrier Poor interactions with healthcare staff Inadequate education delivery Fatalistic Beliefs/Religion *Enablers:* Family and friend support	CASP: 5/10 Rated: Fair

ACE, acute coronary syndrome; CABG, coronary artery bypass graft; PCI, percutaneous coronary intervention; MI , myocardial infarction.

### Theme 1: the relationship between communication and knowledge

Language differences led to ineffective communication, lack of service user knowledge, and reduced CR participation. Eleven out of 13 studies^34,36^ identified language or communication difficulties as a barrier to CR. Studies highlighted multiple contributing factors to communication challenges, such as patients’ limited ability to understand English, clinicians’ inability to speak alternative languages, lack of interpreter availability, reliance on family members for interpretation and insufficient translated written materials.^25,33,35,37,41^

Studies suggest that interpreters can improve communication,^26,27,33,35,39^ although their effectiveness varied. When interpreters were unavailable, family members were often expected to relay important health information.^33,35,38,39^ When clinicians used interpreters or family members for relaying information, information about the clinical diagnosis was prioritized, meaning other topics may be omitted.^33,39^ There were contrasting findings on the usefulness of written materials. Multiple studies identified a need for increased translated written materials to aid communication^24,27,41^; however, other studies identified low literacy levels or a lack of interest in reading these materials reduced their effectiveness.^26,27,39^

Communication barriers were associated with reduced knowledge about heart disease, and the structure, purpose and benefits of CR.^24–27,35,37–41^ Participants who experienced language barriers often displayed a poor understanding of the causes of their cardiac event^25,33,35^ which may have diminished the relevance of attending CR to prevent further cardiac events. Language was highlighted as a direct obstacle to accessing and comprehending information about CR programmes, meaning the benefits of CR and importance of participating to aid recover may have been lost.^24,26,27,37–41^ Knowledge attainment is influenced by a broad range of contributors and was also linked to factors such as timing and method of information delivery, as well as health literacy levels. Participants expressed a preference for verbal information delivered face to face, as this approach felt more personalized and allowed opportunities for clarification.^26,33,34,38,39^ However, these preferences were often not met, and participants did not receive information in the format that was best suited to their needs.^26,38,39^ Low health literacy, compounded by language barriers, was found to discourage CR participation.^39^

### Theme 2: motivation to attend cardiac rehabilitation or follow medical advice

Motivation directly impacted CR participation, and was influenced by a broad range of factors including family and peer support, female sex and health beliefs around heart disease.

All studies in the review found attitudes and motivations towards exercise impacted CR participation with five studies^24,33,39–41^ identifying a lack of interest in exercise or motivation for physical activity as a deterrent to joining a CR programme, regardless of the mode of delivery.

For some participants, their family and peer support effectively motivated them to attend education and exercise classes, and adherence to diet and medication advice.^33,34,39^ The drivers behind how and why family and peer support was provided varied across the studies. Dilla *et al*.,^36^ noted that participants received good support from family members who had also experienced a cardiac event, with the shared experience fostering family bonds. However, some participants reported a lack of motivation to attend CR despite full support from family and friends.^33^ In some cases, family members were concerned about exercise intensity and the volume of recommended lifestyle changes, which discouraged participants from engaging.^34,36,37^ Other external contributors which influenced motivation was mode of delivery. Many participants were motivated by the in-person aspect of centre-based CR,^24,40^ providing an opportunity for socialization and a more interactive experience. Jolly *et al*.^24^ found that those who changed from home-based to centre-based CR due to low motivation were more likely to complete their centre-based CR programme. In-person CR may be the most suitable mode of delivery for some of this population, and the socialization aspect should be promoted to aid participation.

Conflict between clinical advice and some cultural norms related to female sex was evident. South Asian women lacked motivation to engage with home-based programmes, but also lacked confidence to attend the group-based programmes due to unfamiliarity with gym environments and equipment such as weights and treadmills.^25,27,35,40^ Female family members often motivated male relatives to participate in CR,^35,40^ indicating that females are aware of the benefits of CR; however, home duties were often prioritized over their own health, a cultural norm within South Asian communities. In some cases, females were discouraged from attending CR, due to family concerns about balancing participation with family responsibilities.^33,36,37^

Motivation to exercise was closely linked to health beliefs around heart disease.^26,27,36,40^ Participants believed heart disease would result in long-term limitations on physical activity and had poor expectations of what they would gain through being physically active and attending CR.^40,41^ Participants with multiple long-term conditions were less likely to be physically active^24–26,35^ leading to decreased motivation to attend the programme, or adhere to exercise and dietary advice.

### Theme 3: religion

The concepts of religion and fatalism were explored in seven studies. Religion can also influence aspects of daily culture explored in Theme 2; however, the unique aspect of fatalism was considered separately. Some participants explained how their health was ‘Gods will’ or a result of fate, with their own behaviours having little effect on health outcomes.^25,35–37,41^ Despite this, many of these participants still attended CR and did not express that fatalism was a definitive deterrent to participating in CR. In combination with fatalism, previous family history lead to participants expecting to experience a cardiac event and believed that there was little they could do to prevent it.^25,26,36^ These two concepts were often reported together, strengthening the idea that some participants feel they cannot control their health outcomes, meaning they are less likely to attend CR. In contrast, other participants believed they had full control and influence over their health, and these thoughts existed alongside their religious beliefs/faith.^24,26,36,37^ Faith and religion were occasionally prioritized over health behaviours, with CR participation unlikely if the programme interfered with religious commitments such as attending prayer or fasting.^26,35,40^ Some participants identified clinicians’ understanding of religion and its impact on CR attendance as a key enabler.^34^ They felt encouraged and supported when clinicians accommodated religious practices, such as offering alternative class times for prayer or modifying exercises during fasting. This underscores the importance of staff being knowledgeable and compassionate in addressing religious considerations to enhance participation.

### Theme 4: programme delivery

South Asian service users report various barriers related to a lack of culturally appropriate programme delivery. Education and exercise provision that were not culturally focussed led participants to view staff as culturally incompetent, which reduced participation rates and increased attrition.^35,37,39^ Participants expressed that culturally significant topics, such as food preparation and diet, should be informed by appropriate advisory groups and delivered by culturally knowledgeable clinicians to positively impact CR engagement.^33,37,39,40^ Effective communication was again highlighted in relation to personalized programme delivery and cultural competence.^26,33,34,38,39^ Multilingual CR clinicians, women only classes, and programmes that adapted exercises to accommodate traditional clothing and footwear were viewed as inclusive and aided uptake and completion.

Negative experiences when interacting with healthcare professionals reduced CR engagement.^26,35,38–41^ Participants experienced a variety of prejudice, stigma and racism in relation to their South Asian heritage, age, and sex.^35,38,39^ Negative experiences were often linked to communication difficulties, with patients feeling excluded if they did not speak English. In contrast, supportive and friendly interactions with healthcare staff positively impacted CR participation and motivated patients to adhere to the programme.^34^

Specific to South Asian populations, participants indicated that flexible class timings and modes of delivery enabled them to fit CR around their cultural commitments such as prayer.^35,40^ This unintentional culturally relevant outcome of programme delivery highlights how some aspects of CR programmes may already support participation for this ethnic group. Modes such as home-based CR provided convenience and centre-based CR provided a supervised exercise element, and the choice of delivery was empowering, providing a tailored experience and increased participation.^24,34,38,40^ This was particularly relevant for those who were employed,^25,35^ an aspect of programme delivery pertinent to South Asian service users who experience CVD at a younger age^21^ and are more likely to balance CR attendance with employment commitments. Participants perceived centre-based supervised exercise programmes as safe, as activity was prescribed and monitored, and a main reason behind choosing to attend centre-based CR.^24,26,34^

### Theme 5: practical considerations

A lack of access to transport,^27,34,35,37^ unsuitable class timings^24,35,37,38,40^ and classes located far from home^24,40^ all acted as barriers. Participants indicated that transport to and from CR was provided by family members, taxi services, or different forms of public transport.^24,27,35,37,38^ Employment and financial concerns were also expressed in several studies,^25–27,35,37,38^ with patients finding it difficult to balance work commitments and attending CR. Often, due to financial constraints, work was a priority.

## Discussion

This review provides a comprehensive analysis of barriers and enablers to CR participation after referral, that are either prominent or unique to South Asian CR service users when they are in an ethnic minority. Many barriers have been reported by other ethnicities such as transport and programme delivery,^18,42^ however, some appear to be specific to South Asian service users when compared with White Europeans, including language, fatalism and religion, and females prioritizing home duties.^23,42,43^

The review found a need for early and effective communication, delivered verbally and face to -face, with qualified translators. Similar findings were presented in other scoping and narrative reviews which included other ethnic minorities.^15,23,44^ The link between effective communication and increased knowledge has the potential to drive behaviour change and increase CR participation,^20,45^ however, this does not always guarantee behaviour change. Knowledge is rarely sufficient by itself, and a multi-factorial approach incorporating psychosocial contexts, values, and beliefs is needed for clinicians to elicit desired actions.^46^ Studies in this review surmised that effective education about the benefits of CR is a key enabler to CR enrolment,^34,35,38–41,44^ however, this approach cannot be considered in silo. Motivation is a key driver of developing healthy lifestyle behaviours. It has been suggested that South Asian individuals rely heavily on extrinsic motivational factors.^2^ which are interwoven with South Asian societal and cultural norms. Multiple sources of extrinsic motivation such as family and peer support, and positive healthcare experiences were identified. Cardiac rehabilitation participation may be more successful if clinicians understood service users culturally sensitive motivational drivers of CR engagement, alongside increasing effective communication and CVD knowledge.^46^ The emphasis on enhancing motivational triggers may be a key future focus as the health needs of the South Asian population become more complex.

The South Asian diaspora experience barriers and enablers at patient, clinician and systemic level when attempting to engage with a system not designed with their societal and cultural norms in mind. South Asian service users face similar barriers to other ethnicities,^18^ however these are not always experienced in equal measures. The presence of multiple long-term conditions is reported as a barrier to CR across multiple ethnicities, however South Asian service users live with higher numbers of co-morbidities compared with other ethnicities,^47^ therefore this barrier may be amplified in South Asian service users. Additionally, some barriers are more unique to South Asian service users such as religion and fatalism. Ninety-two per cent of British Asians have a religion compared with 58% White British^48^ and strong religious beliefs influence use of healthcare services and health outcomes.^49^ Fatalism can reduce engagement in other lifestyle behaviours such as medication adherence and diet improvements, all important elements of CR.^50,51^ Addressing fatalistic beliefs, and aiming for a balance between respecting religious beliefs and advising according to clinical guidelines, may improve CR participation and engagement with other health-related behaviours associated with CHD risk factors.^51^

The review identifies important implications for practice. It is imperative that clinicians comprehend how culture and religion influence motivation and health-related behaviours for South Asian service users. Effective use of professional interpreters and translated written materials must be a priority and not disregarded due to cost or resource. Healthcare organizations must take responsibility to ensure culturally competent clinicians deliver clinical care congruent with service users’ societal and cultural norms, and it is essential they are proactive in addressing stigma and racism across all healthcare interactions. Health inequalities are often shaped by intersecting social identities such as ethnicity and SES, which together can amplify disparities in healthcare engagement. In the UK, South Asians are more likely to live in the most deprived neighbourhoods compared with White British, are twice as likely to be in the bottom fifth of incomes and are regularly employed in low-pay and insecure workplaces.^52–54^ Given that individuals from lower SES backgrounds and from an ethnic minority are already less likely to attend CR,^55^ the intersection of these factors exacerbates this issue, and may result in even lower uptake and completion rates. It is crucial for healthcare clinicians to consider such intersection when providing care or redesigning services to ensure they are accessible, effective, and equitable for their local population. The current CR operates an equal distribution of services to all its participants, unintentionally resulting in unequal access and outcomes for ethnic minorities. To deliver an equitable service, an unequal distribution of services is needed. Identifying barriers and enablers is not enough to elicit engagement; however, understanding unique, ethnic specific barriers and enablers is the first step towards redistributing resources and providing equitable services.

This comprehensive review highlights important barriers and enablers of CR in minority South Asian communities. In relation to sex, the results are generalizable to a broader South Asian CVD population, with 68% of participants being male, representative of the distribution of CVD within this ethnic minority group.^36^ However, there are limitations. The majority of studies had a ‘fair’ quality appraisal score. Most participants were CR attendees, which influenced the depth of data regarding barriers to uptake and findings cannot be extrapolated to referral. Most studies conducted semi-structured interviews which are subject to bias and low validity, and their quality is dependent on the skill and experience of the interviewer. Not all studies identified which stage of CR the barriers apply to and the grouping of South Asian service users did not allow for in-depth exploration of cultural differences among different South Asian communities. Research from only two countries was identified for this review, representing a narrow geographical sample, therefore, the findings cannot be generalized to other countries that may have different healthcare systems.

Further research that accurately records and reports ethnicity while capturing cultural diversity within each group is essential for developing a more comprehensive understanding of individual needs both between and within populations, and could be used to inform targeted programme adaptations that ensure equitable CR delivery. Once these adaptations are implemented, ongoing evaluation is necessary to measuring their impact on uptake, completion, and effectiveness to ensure sustained improvements in participation and clinical outcomes.

## Conclusion

To enhance CR participation among South Asian individuals, prominent barriers must be addressed and enablers strengthened. Support from family, targeting motivational triggers, effective communication and positive healthcare interactions with culturally competent clinicians could serve as key enablers. While not all barriers and enablers stem from a participant’s ethnicity, it is essential for clinicians to identify barriers pertinent to South Asian individuals and utilize this knowledge to tailor services to individual needs.

## Supplementary Material

zvaf044_Supplementary_Data

## Data Availability

No new data were generated or analysed in support of this research.
