# Supplementary material for: Identifying barriers and enablers to cardiac rehabilitation participation and completion unique to South Asian individuals: a qualitative systematic review
Source: Eur J Cardiovasc Nurs. 2025 Mar 21;24(5):670–85. doi: 10.1093/eurjcn/zvaf044 (PMC12278909; doi:10.1093/eurjcn/zvaf044)
Supplement: zvaf044_Supplementary_Data [file zvaf044_supplementary_data.pdf]

Supplementary File

Identifying Barriers and Enablers to Cardiac Rehabilitation Participation and Completion Unique to South Asian Individuals: A Systematic Review of Global Evidence

Table of Contents

Contents

S1 Table: Database Search Results 2

Database: Ovid MEDLINE(R) ALL 1946 to January 16, 2024 2

Database: Web of Science 5

Database: Cochrane Central Register of Controlled Trials (CENTRAL) 6

Database: Pubmed 8

Database: CINAHL Plus with Full Text 9

Database: APA PsychInfo with Full Text 11

S2 Table: PRISMA Systematic Review Checklist 15

S3 Table: The ENTREQ Checklist 18

Supplementary tables

S1 Table: Database Search Results

Database: Ovid MEDLINE(R) ALL 1946 to January 16, 2024
Search Strategy:

#	Searches	Results
1	Exercise Therapy.mp. or exp Exercise Therapy/	70868
2	Cardiac Rehabilitation.mp. or exp Cardiac Rehabilitation/	10578
3	"cardiac rehab*".mp.	10625
4	"Cardiovascular rehab*".mp.	437
5	"Cardiac rehabilitation".mp.	10578
6	"Exercise Therapy".mp.	55458
7	"Rehabilitation Nursing".mp.	2073
8	"Heart Rehabilitation".mp.	33
9	"Physical Rehab*".mp.	2952
10	"Rehab* Centre".mp.	1577
11	"Rehab* training".mp.	2747
12	"cardiovascular exercise".mp.	296
13	"cardiovascular disease rehab*".mp.	5
14	1 or 2 or 3 or 4 or 5 or 6 or 7 or 8 or 9 or 10 or 11 or 12 or 13	87949
15	South Asian People.mp. or exp South Asian People/	330
16	"south asia*".mp.	14662
17	exp Asia, Western/	354922
18	"south india*".mp.	10551
19	Bangladesh*.mp.	25363
20	Bengal*.mp.	13141
21	Gujerat*.mp.	20
22	Gujarat*.mp.	2521
23	Punjab*.mp.	5382
24	Hindu*.mp.	2723
25	Pakistan*.mp.	40773
26	Tamil*.mp.	4638
27	"Sri Lanka*".mp.	11872
28	Ceylon*.mp.	834

29	Sinhal*.mp.	467
30	Sikh*.mp.	480
31	Nepal*.mp.	18277
32	Hindi*.mp.	6987
33	Urdu*.mp.	771
34	Pathan.mp.	79
35	pushto.mp.	9
36	Syleti*.mp.	1
37	Jain*.mp.	668
38	khoja.mp.	8
39	eelam.mp.	5
40	Telugu*.mp.	111
41	Marath*.mp.	4982
42	Kannad.mp.	2
43	Malayala*.mp.	174
44	Oriya*.mp.	16
45	Andhra*.mp.	2202
46	Kerala*.mp.	3851
47	Karnatak*.mp.	3190
48	Assam*.mp.	2471
49	Bihar*.mp.	1989
50	Uttar*.mp.	3619
51	Himachal*.mp.	1178
52	Kashmir*.mp.	2544
53	Pushtun.mp.	2
54	Maharashtrian*.mp.	47
55	Parsi*.mp.	19307
56	Sylheti*.mp.	33
57	Gujar*.mp.	2549
58	Gujjar*.mp.	61
59	Gurjar*.mp.	12
60	Goojar*.mp.	0
61	Gujur*.mp.	13
62	Gojri*.mp.	4

63	Gujari*.mp.	0
64	Gujrat*.mp.	111
65	Bhutan*.mp.	1538
66	"South east asia*".mp.	6377
67	"british india*".mp.	145
68	ethnic.mp.	114831
69	immigra*.mp.	64497
70	"south asain indo*".mp.	0
71	(India* adj5 muslim*).mp.	216
72	(India* adj5 islam*).mp.	241
73	"ethnic* minorit*".mp.	17971
74	15 or 16 or 17 or 18 or 19 or 20 or 21 or 22 or 23 or 24 or 25 or 26 or 27 or 28 or 29 or 30 or 31 or 32 or 33 or 34 or 35 or 36 or 37 or 38 or 39 or 40 or 41 or 42 or 43 or 44 or 45 or 46 or 47 or 48 or 49 or 50 or 51 or 52 or 53 or 54 or 55 or 56 or 57 or 58 or 59 or 60 or 61 or 62 or 63 or 64 or 65 or 66 or 67 or 68 or 69 or 70 or 71 or 72 or 73	616695
75	14 and 74	828

Database: Web of Science

7	#4 AND #6 and Preprint Citation Index (Exclude – Database)	805
6	(TS=("South Asian People")) OR TS=(Asia, Western)) OR TS=("South Asia*")) OR TS=("south india*")) OR TS=(Bangladesh*))) OR TS=(Bengal* OR Gujerat* OR Gujarat* OR Punjab* OR Hindu* OR Pakistan* OR Tamil* OR "Sri Lanka*" OR Ceylon* OR Sinhal* OR Sikh* OR Nepal* OR Hindi* OR Urdu* OR patran OR pashto OR sylheti OR Jain* OR khola OR eslam OR Telugu* OR Marath* OR Kannad* OR Malayala* OR Oriya* OR Andhra* OR Kerala* OR Karnatak* OR Assam* OR Bihar* OR Uttar* OR Himachal* OR Kashmir* OR pashtun or Maharashtrian* OR Parsi* OR Sylheti* OR Gujar* OR Gujjar* OR Gurjar* OR Goojar* OR Gujur* OR Gojri* OR Gujari* OR Gujrat* OR Bhutan* OR "south asia*" OR “South east asia*” OR "british india*" OR ethnic OR "ethnic* minorit*" OR immigra* OR “south asain indo*") and Preprint Citation Index (Exclude – Database)	1,575, 188
	(TS=("South Asian People")) OR TS=(Asia, Western)) OR TS=("South Asia*")) OR TS=("south india*")) OR TS=(Bangladesh*))) OR TS=(Bengal* OR Gujerat* OR Gujarat* OR Punjab* OR Hindu* OR Pakistan* OR Tamil* OR "Sri Lanka*" OR Ceylon* OR Sinhal* OR Sikh* OR Nepal* OR Hindi* OR Urdu* OR Pathan OR pushto OR Syleti OR Jain* OR khoja OR eelam OR Telugu* OR Marath* OR Kannad* OR Malayala* OR Oriya* OR Andhra* OR Kerala* OR Karnatak* OR Assam* OR Bihar* OR Uttar* OR Himachal* OR Kashmir* OR Pushtun or Maharashtrian* OR Parsi* OR Sylheti* OR Gujar* OR Gujjar* OR Gurjar* OR Goojar* OR Gujur* OR Gojri* OR Gujari* OR Gujrat* OR Bhutan* OR "south asia*" OR “South east asia*” OR "british india*" OR ethnic OR "ethnic* minorit*" OR immigra* OR “south asain indo*") and Preprint Citation Index (Exclude – Database)	1,574,355
4	#1 OR #2 OR #3 and Preprint Citation Index (Exclude – Database)	91,325
3	(TS=("Exercise Therapy")) OR TS=("Cardiovascular rehab*")) OR TS=(“Rehab* Nursing”)) OR TS=(“Heart Rehab*”)) OR TS=(“Physical Rehab*”)) OR TS=(“Rehab*Centre”)) OR TS=(“Rehab* training”)) OR TS=(“cardiovas* exercise”)) OR TS=(“cardiovascular disease rehab*”) and Preprint Citation Index (Exclude – Database)	77,019
2	TS=("Exercise Therapy") and Preprint Citation Index (Exclude – Database)	64,087
1	"cardiac rehab*" (Topic) and Preprint Citation Index (Exclude – Database)	17,472

Database: Cochrane Central Register of Controlled Trials (CENTRAL)

#1	MeSH descriptor: [Cardiac Rehabilitation] explode all trees	622
#2	("cardiac rehabilitation"):ti,ab,kw (Word variations have been searched)	3471
#3	(Exercise Therapy):ti,ab,kw (Word variations have been searched)	65012
#4	(Rehabilitation Nursing):ti,ab,kw (Word variations have been searched)	3893
#5	(heart rehabilitation):ti,ab,kw (Word variations have been searched)	7186
#6	(Physical Rehabilitation):ti,ab,kw (Word variations have been searched)	21419
#7	(Rehabilitation training):ti,ab,kw (Word variations have been searched)	24758
#8	(cardiovascular exercise):ti,ab,kw (Word variations have been searched)	13847
#9	(cardiovascular disease rehabilitation):ti,ab,kw (Word variations have been searched)	2220
#10	MeSH descriptor: [Exercise Therapy] explode all trees	22405
#11	#1 OR #10	22800
#12	#2 OR #3 OR #4 OR #5 OR #6 OR #7 OR #8 OR #9	101771
#13	#11 OR #12	105679
#14	MeSH descriptor: [Asia, Western] explode all trees	11933
#15	(south india):ti,ab,kw (Word variations have been searched)	1013
#16	(Bangladesh*):ti,ab,kw (Word variations have been searched)	2350
#17	(Bengal*):ti,ab,kw (Word variations have been searched)	749
#18	(Gujerat*):ti,ab,kw (Word variations have been searched)	0
#19	(Gujarat*):ti,ab,kw (Word variations have been searched)	229
#20	(Punjab*):ti,ab,kw (Word variations have been searched)	302
#21	(Hindu*):ti,ab,kw (Word variations have been searched)	100
#22	(Pakistan*):ti,ab,kw (Word variations have been searched)	3107
#23	(Tamil*):ti,ab,kw (Word variations have been searched)	372
#24	(Sri Lanka*):ti,ab,kw (Word variations have been searched)	729
#25	(Ceylon*):ti,ab,kw (Word variations have been searched)	15
#26	(Sinhal*):ti,ab,kw (Word variations have been searched)	51
#27	(Sikh*):ti,ab,kw (Word variations have been searched)	6
#28	(Nepal*):ti,ab,kw (Word variations have been searched)	1233
#29	(Hindi*):ti,ab,kw (Word variations have been searched)	643
#30	(Urdu*):ti,ab,kw (Word variations have been searched)	126
#31	(Pathan):ti,ab,kw (Word variations have been searched)	1
#32	(pushto):ti,ab,kw (Word variations have been searched)	1
#33	(Syleti):ti,ab,kw (Word variations have been searched)	0
#34	(Jain*):ti,ab,kw (Word variations have been searched)	83
#35	(khoja):ti,ab,kw (Word variations have been searched)	3
#36	(eelam):ti,ab,kw (Word variations have been searched)	1
#37	(Telugu):ti,ab,kw (Word variations have been searched)	21
#38	(Marath*):ti,ab,kw (Word variations have been searched)	470
#39	(Kannad*):ti,ab,kw (Word variations have been searched)	187
#40	(Malayala*):ti,ab,kw (Word variations have been searched)	60
#41	(Oriya*):ti,ab,kw (Word variations have been searched)	4
#42	(Andhra*):ti,ab,kw (Word variations have been searched)	124
#43	(Kerala*):ti,ab,kw (Word variations have been searched)	248
#44	(Karnatak*):ti,ab,kw (Word variations have been searched)	302
#45	(Assam*):ti,ab,kw (Word variations have been searched)	66
#46	(Bihar*):ti,ab,kw (Word variations have been searched)	306
#47	(Uttar*):ti,ab,kw (Word variations have been searched)	288
#48	(Himachal*):ti,ab,kw (Word variations have been searched)	28
#49	(Kashmir*):ti,ab,kw (Word variations have been searched)	76
#50	(Pushtun):ti,ab,kw (Word variations have been searched)	0

#51	(Maharashtrian*):ti,ab,kw (Word variations have been searched)	1
#52	(Parsi*):ti,ab,kw (Word variations have been searched)	497
#53	(Sylheti*):ti,ab,kw (Word variations have been searched)	6
#54	(Gujar*):ti,ab,kw (Word variations have been searched)	235
#55	(Gujjar*):ti,ab,kw (Word variations have been searched)	0
#56	(Gurjar*):ti,ab,kw (Word variations have been searched)	0
#57	(Goojar*):ti,ab,kw (Word variations have been searched)	0
#58	(Gujur*):ti,ab,kw (Word variations have been searched)	1
#59	(Gojri*):ti,ab,kw (Word variations have been searched)	0
#60	(Gujari*):ti,ab,kw (Word variations have been searched)	0
#61	(Gujrat*):ti,ab,kw (Word variations have been searched)	30
#62	(Bhutan*):ti,ab,kw (Word variations have been searched)	40
#63	(south asia*):ti,ab,kw (Word variations have been searched)	2272
#64	(South east asia*):ti,ab,kw (Word variations have been searched)	459
#65	(british india*):ti,ab,kw (Word variations have been searched)	40
#66	(ethnic* minorit*):ti,ab,kw (Word variations have been searched)	2303
#67	(immigra*):ti,ab,kw (Word variations have been searched)	1200
#68	(south asain indo*):ti,ab,kw (Word variations have been searched)	0
#69	#14 OR #15 OR #16 OR #17 OR #18 OR #19 OR #20 OR #21 OR #22 OR #23 OR #24 OR #25 OR #26 OR #27 OR #28 OR #29 OR #30 OR #31 OR #32 OR #33 OR #35 OR #36 OR #37 OR #38	19483
#70	#39 OR #40 OR #41 OR #42 OR #43 OR #44 OR #45 OR #46 OR #47 OR #48 OR #49 OR #50 OR #51 OR #52 OR #53 OR #54 OR #55 OR #56 OR #57 OR #58 OR #59 OR #60 OR #61 OR #62 OR #63 OR #64 OR #65 OR #66 OR #67 OR #68	8035
#71	#69 OR #70	26088
#72	#13 AND 71	559

Database: Pubmed

Total: 350 16th August 2023

((((((((((((("cardiac rehab*"[Text Word]) OR ("Cardiovascular rehab*"[Text Word])) OR ("Cardiac rehabilitation"[Text Word])) OR ("Exercise Therapy"[Text Word])) OR ("Rehabilitation Nursing"[Text Word])) OR ("Heart Rehabilitation"[Text Word])) OR ("Physical Rehabilitation"[Text Word])) OR ("Rehabilitation Centre"[Text Word]))) OR ("Rehabilitation training"[Text Word])) OR ("cardiovascular exercise"[Text Word])))) AND ((("south india*"[Text Word] OR Bangladesh*[Text Word] OR Bengal*[Text Word] OR Gujerat*[Text Word] OR Gujarat*[Text Word] OR Punjab*[Text Word] OR Hindu*[Text Word] OR Pakistan*[Text Word] OR Tamil*[Text Word] OR "Sri Lanka*"[Text Word] OR Ceylon*[Text Word] OR Sinhal*[Text Word] OR Sikh*[Text Word] OR Nepal*[Text Word] OR Hindi*[Text Word] OR Urdu*[Text Word] OR Pathan[Text Word] OR pushto[Text Word] OR Syleti[Text Word] OR Jain*[Text Word] OR khoja[Text Word] OR eelam[Text Word] OR Telugu*[Text Word] OR Marath*[Text Word] OR Kannad*[Text Word] OR Malayala*[Text Word] OR Oriya*[Text Word] OR Andhra*[Text Word] OR Kerala*[Text Word] OR Karnatak*[Text Word] OR Assam*[Text Word] OR Bihar*[Text Word] OR Uttar*[Text Word] OR Himachal*[Text Word] OR Kashmir*[Text Word] OR Pushtun[Text Word] OR Maharashtrian*[Text Word] OR Parsi* Sylheti*[Text Word] OR Gujar*[Text Word] OR Gujjar*[Text Word] OR Gurjar*[Text Word] OR Goojar*[Text Word] OR Gujur*[Text Word] OR Gojri*[Text Word] OR Gujari*[Text Word] OR Gujrat*[Text Word] OR Bhutan*[Text Word] OR "south asia*"[Text Word] OR Southeastasia*[Text Word] OR "british india*"[Text Word] OR ethnic[Text Word] OR immigr*[Text Word] OR "south asain indo*[Text Word] OR India* adj5 muslim*[Text Word] OR India* adj5 islam*[Text Word]))))

Database: CINAHL Plus with Full Text

#	Query	Limiters/Expanders	Results
S10	S4 AND S9	Expanders - Apply equivalent subjects<br>Search modes - Proximity	69
S9	S5 OR S6 OR S7 OR S8	Expanders - Apply equivalent subjects<br>Search modes - Proximity	153,672
S8	"south india*" OR Bangladesh* OR Bengal* OR Gujerat* OR Gujarat* OR Punjab* OR Hindu* OR Pakistan* OR Tamil* OR "Sri Lanka*" OR Ceylon* OR Sinhal* OR Sikh* OR Nepal* OR Hindi* OR Urdu* OR Pathan OR pushto OR Syleti OR Jain* OR khoja OR eelam OR Telugu* OR Marath* OR Kannad* OR Malayala* OR Oriya* OR Andhra* OR Kerala* OR Karnatak* OR Assam* OR Bihar* OR Uttar* OR Himachal* OR Kashmir* OR Pushtun or Maharashtrian* OR Parsi* OR Sylheti* OR Gujar* OR Gujjar* OR Gurjar* OR Goojar* OR Gujur* OR Gojri* OR Gujari* OR Gujrat* OR Bhutan* OR "south asia*" OR “South east asia*” OR "british india*" OR ethnic OR "ethnic* minorit*" OR immigra* OR “south asain indo*	Expanders - Apply equivalent subjects<br>Search modes - Proximity	153,500
S7	MW Asia, Western	Expanders - Apply equivalent subjects<br>Search modes - Proximity	292
S6	MW South Asia*	Expanders - Apply equivalent subjects<br>Search modes - Proximity	135
S5	MW South Asian People	Expanders - Apply equivalent subjects<br>Search modes - Proximity	0
S4	S1 OR S2 OR S3	Expanders - Apply equivalent subjects<br>Search modes - Proximity	7,270
S3	MW ("cardiac rehab*" OR "Cardiovascular rehab*" OR “Cardiac rehabilitation” OR “Exercise Therapy” OR “Rehab* Nursing” OR “Heart Rehab*” OR “Physical Rehab*” OR “Rehab*Centre” OR “Rehab* training” OR “cardiovas* exercise” OR “cardiovascular disease rehab*”)	Expanders - Apply equivalent subjects<br>Search modes - Proximity	2,016
S2	MW Exercise Therapy	Expanders - Apply equivalent subjects<br>Search modes - Proximity	206

S1	MW (cardiac rehabilitation or cardiovascular rehabilitation or cardiac rehab or cardiovascular rehab)	Expanders - Apply equivalent subjects<br>Search modes - Proximity	5,127

Database: APA PsychInfo with Full Text

S79	S16 AND S78	Expanders - Apply equivalent subjects<br>Search modes - Proximity	20
S78	S17 OR S18 OR S19 OR S20 OR S21 OR S22 OR S23 OR S24 OR S25 OR S26 OR S27 OR S28 OR S29 OR S30 OR S31 OR S32 OR S33 OR S34 OR S35 OR S36 OR S37 OR S38 OR S39 OR S40 OR S41 OR S42 OR S43 OR S44 OR S45 OR S46 OR S47 OR S48 OR S49 OR S50 OR S51 OR S52 OR S53 OR S54 OR S55 OR S56 OR S57 OR S58 OR S59 OR S60 OR S61 OR S62 OR S63 OR S64 OR S65 OR S66 OR S67 OR S68 OR S69 OR S70 OR S71 OR S72 OR S73	Expanders - Apply equivalent subjects<br>Search modes - Proximity	67,268
S77	KW India* adj5 islam*	Expanders - Apply equivalent subjects<br>Search modes - Proximity	0
S76	KW India* adj5 islam*).	Expanders - Apply equivalent subjects<br>Search modes - Proximity	0
S75	KW India* adj5 muslim*	Expanders - Apply equivalent subjects<br>Search modes - Proximity	0
S74	KW “south asain indo*"	Expanders - Apply equivalent subjects<br>Search modes - Proximity	0
S73	KW immigra*	Expanders - Apply equivalent subjects<br>Search modes - Proximity	23,376
S72	KW "ethnic* minorit*"	Expanders - Apply equivalent subjects<br>Search modes - Proximity	4,480
S71	KW ethnic*	Expanders - Apply equivalent subjects<br>Search modes - Proximity	37,319
S70	KW "british india*"	Expanders - Apply equivalent subjects<br>Search modes - Proximity	23
S69	KW “South east asia*”	Expanders - Apply equivalent subjects<br>Search modes - Proximity	76
S68	KW "south asia*"	Expanders - Apply equivalent subjects<br>Search modes - Proximity	1,310
S67	KW Bhutan*	Expanders - Apply equivalent subjects<br>Search modes - Proximity	133
S66	KW Gujjar*	Expanders - Apply equivalent subjects<br>Search modes - Proximity	0
S65	KW Gujar*	Expanders - Apply equivalent subjects<br>Search modes - Proximity	67
S64	KW Sylheti*	Expanders - Apply equivalent subjects<br>Search modes - Proximity	13
S63	KW Parsi*	Expanders - Apply equivalent subjects<br>Search modes - Proximity	720
S62	KW Maharashtrian*	Expanders - Apply equivalent subjects<br>Search modes - Proximity	1
S61	KW Pushtun	Expanders - Apply equivalent subjects<br>Search modes - Proximity	0

S60	KW Kashmir*	Expanders - Apply equivalent subjects<br>Search modes - Proximity	81
S59	KW Himachal*	Expanders - Apply equivalent subjects<br>Search modes - Proximity	2
S58	KW Uttar*	Expanders - Apply equivalent subjects<br>Search modes - Proximity	22
S57	KW Bihar*	Expanders - Apply equivalent subjects<br>Search modes - Proximity	23
S56	KW Assam*	Expanders - Apply equivalent subjects<br>Search modes - Proximity	35
S55	KW Karnatak*	Expanders - Apply equivalent subjects<br>Search modes - Proximity	10
S54	KW Kerala*	Expanders - Apply equivalent subjects<br>Search modes - Proximity	60
S53	KW Andhra*	Expanders - Apply equivalent subjects<br>Search modes - Proximity	6
S52	KW Oriya*	Expanders - Apply equivalent subjects<br>Search modes - Proximity	21
S51	KW Malayala*	Expanders - Apply equivalent subjects<br>Search modes - Proximity	23
S50	KW Kannad*	Expanders - Apply equivalent subjects<br>Search modes - Proximity	71
S49	KW Marath*	Expanders - Apply equivalent subjects<br>Search modes - Proximity	464
S48	KW Telugu*	Expanders - Apply equivalent subjects<br>Search modes - Proximity	16
S47	KW eelam	Expanders - Apply equivalent subjects<br>Search modes - Proximity	5
S46	KW khoja	Expanders - Apply equivalent subjects<br>Search modes - Proximity	2
S45	KW Jain*	Expanders - Apply equivalent subjects<br>Search modes - Proximity	31
S44	KW Syleti*	Expanders - Apply equivalent subjects<br>Search modes - Proximity	0
S43	KW Syleti	Expanders - Apply equivalent subjects<br>Search modes - Proximity	0
S42	KW pusht*	Expanders - Apply equivalent subjects<br>Search modes - SmartText Searching	0
S41	KW pusht*	Expanders - Apply equivalent subjects<br>Search modes - Proximity	0
S40	KW pushto*	Expanders - Apply equivalent subjects<br>Search modes - Proximity	0
S39	KW pushto*	Expanders - Apply equivalent subjects<br>Search modes - SmartText Searching	0

S38	KW pushto*	Expanders - Apply equivalent subjects<br>Search modes - Proximity	0
S37	KW pushto	Expanders - Apply equivalent subjects<br>Search modes - Proximity	0
S36	KW Pathan	Expanders - Apply equivalent subjects<br>Search modes - Proximity	3
S35	KW Urdu*	Expanders - Apply equivalent subjects<br>Search modes - Proximity	111
S34	KW Hindi*	Expanders - Apply equivalent subjects<br>Search modes - Proximity	336
S33	KW Nepal*	Expanders - Apply equivalent subjects<br>Search modes - Proximity	803
S32	KW Sikh*	Expanders - Apply equivalent subjects<br>Search modes - Proximity	148
S31	KW Sinhal*	Expanders - Apply equivalent subjects<br>Search modes - Proximity	43
S30	KW Ceylon*	Expanders - Apply equivalent subjects<br>Search modes - Proximity	30
S29	KW "Sri Lanka*"	Expanders - Apply equivalent subjects<br>Search modes - Proximity	645
S28	KW Tamil*	Expanders - Apply equivalent subjects<br>Search modes - Proximity	164
S27	KW Pakistan*	Expanders - Apply equivalent subjects<br>Search modes - Proximity	1,635
S26	KW Hindu*	Expanders - Apply equivalent subjects<br>Search modes - Proximity	1,033
S25	KW Punjab*	Expanders - Apply equivalent subjects<br>Search modes - Proximity	136
S24	KW Gujarat*	Expanders - Apply equivalent subjects<br>Search modes - Proximity	66
S23	KW Gujerat*	Expanders - Apply equivalent subjects<br>Search modes - Proximity	0
S22	KW Bengal*	Expanders - Apply equivalent subjects<br>Search modes - Proximity	260
S21	KW Bangladesh*	Expanders - Apply equivalent subjects<br>Search modes - Proximity	1,250
S20	KW "south india*"	Expanders - Apply equivalent subjects<br>Search modes - Proximity	138
S19	MA Asia, Western	Expanders - Apply equivalent subjects<br>Search modes - Proximity	79
S18	MA South Asia*	Expanders - Apply equivalent subjects<br>Search modes - Proximity	3
S17	MA South Asian People	Expanders - Apply equivalent subjects<br>Search modes - Proximity	3

S16	S1 OR S2 OR S3 OR S4 OR S5 OR S6 OR S7 OR S8 OR S9 OR S10 OR S11 OR S12 OR S13 OR S14 OR S15	Expanders - Apply equivalent subjects<br>Search modes - Proximity	7,007
S15	KW “cardiovascular disease rehab*”	Expanders - Apply equivalent subjects<br>Search modes - SmartText Searching	17
S14	KW “cardiovascular disease rehab*”	Expanders - Apply equivalent subjects<br>Search modes - Proximity	0
S13	KW “cardiovas* exercise”	Expanders - Apply equivalent subjects<br>Search modes - Proximity	45
S12	KW “Rehab* training”	Expanders - Apply equivalent subjects<br>Search modes - Proximity	115
S11	KW “Rehab* Centre”	Expanders - Apply equivalent subjects<br>Search modes - Proximity	27
S10	KW “Rehab*Centre”	Expanders - Apply equivalent subjects<br>Search modes - Proximity	0
S9	KW “Physical Rehab*”	Expanders - Apply equivalent subjects<br>Search modes - Proximity	191
S8	KW “Heart Rehab*”	Expanders - Apply equivalent subjects<br>Search modes - Proximity	1
S7	KW “Rehab* Nursing”	Expanders - Apply equivalent subjects<br>Search modes - Proximity	30
S6	KW “Exercise Therapy”	Expanders - Apply equivalent subjects<br>Search modes - Proximity	303
S5	KW “Cardiac rehabilitation”	Expanders - Apply equivalent subjects<br>Search modes - Proximity	728
S4	KW "Cardiovascular rehab*"	Expanders - Apply equivalent subjects<br>Search modes - Proximity	23
S3	KW "cardiac rehab*"	Expanders - Apply equivalent subjects<br>Search modes - Proximity	731
S2	MA (exercise therapy or physical therapy or physiotherapy or exercise intervention)	Expanders - Apply equivalent subjects<br>Search modes - Proximity	5,666
S1	MA (cardiac rehabilitation or cardiovascular rehabilitation or cardiac rehab or cardiovascular rehab)	Expanders - Apply equivalent subjects<br>Search modes - Proximity	93

S2 Table: PRISMA Systematic Review Checklist

Section and Topic	Item #	Checklist item	Location where item is reported
**TITLE**			
Title	1	Identify the report as a systematic review.	Title page
**ABSTRACT**			
Abstract	2	See the PRISMA 2020 for Abstracts checklist.	Abstract page 1
**INTRODUCTION**			
Rationale	3	Describe the rationale for the review in the context of existing knowledge.	Page 4-7
Objectives	4	Provide an explicit statement of the objective(s) or question(s) the review addresses.	Page 7
**METHODS**			
Eligibility criteria	5	Specify the inclusion and exclusion criteria for the review and how studies were grouped for the syntheses.	Page 7
Information sources	6	Specify all databases, registers, websites, organisations, reference lists and other sources searched or consulted to identify studies. Specify the date when each source was last searched or consulted.	Page 7
Search strategy	7	Present the full search strategies for all databases, registers and websites, including any filters and limits used.	Page 7<br>Supplementary Table S1
Selection process	8	Specify the methods used to decide whether a study met the inclusion criteria of the review, including how many reviewers screened each record and each report retrieved, whether they worked independently, and if applicable, details of automation tools used in the process.	Page 7-9
Data collection process	9	Specify the methods used to collect data from reports, including how many reviewers collected data from each report, whether they worked independently, any processes for obtaining or confirming data from study investigators, and if applicable, details of automation tools used in the process.	Page 7-9
Data items	10a	List and define all outcomes for which data were sought. Specify whether all results that were compatible with each outcome domain in each study were sought (e.g. for all measures, time points, analyses), and if not, the methods used to decide which results to collect.	Page 7-8
	10b	List and define all other variables for which data were sought (e.g. participant and intervention characteristics, funding sources). Describe any assumptions made about any missing or unclear information.	Page 7-8<br>Table 1
Study risk of bias assessment	11	Specify the methods used to assess risk of bias in the included studies, including details of the tool(s) used, how many reviewers assessed each study and whether they worked independently, and if applicable, details of automation tools used in the process.	Page 8<br>Table 2
Effect measures	12	Specify for each outcome the effect measure(s) (e.g. risk ratio, mean difference) used in the synthesis or presentation of results.	Not applicable
Synthesis methods	13a	Describe the processes used to decide which studies were eligible for each synthesis (e.g. tabulating the study intervention characteristics and comparing against the planned groups for each synthesis (item #5)).	Page 8-9<br>Table 1
	13b	Describe any methods required to prepare the data for presentation or synthesis, such as handling of missing summary statistics, or data conversions.	Not applicable
	13c	Describe any methods used to tabulate or visually display results of individual studies and syntheses.	Not applicable
	13d	Describe any methods used to synthesize results and provide a rationale for the choice(s). If meta-analysis was performed, describe the model(s), method(s) to identify the presence and extent of statistical heterogeneity, and software package(s) used.	8-9
	13e	Describe any methods used to explore possible causes of heterogeneity among study results (e.g. subgroup analysis, meta-regression).	Not applicable
	13f	Describe any sensitivity analyses conducted to assess robustness of the synthesized results.	Not applicable

Section and Topic	Item #	Checklist item	Location where item is reported
Reporting bias assessment	14	Describe any methods used to assess risk of bias due to missing results in a synthesis (arising from reporting biases).	Not applicable
Certainty assessment	15	Describe any methods used to assess certainty (or confidence) in the body of evidence for an outcome.	Page 8<br>Table 2
**RESULTS**			
Study selection	16a	Describe the results of the search and selection process, from the number of records identified in the search to the number of studies included in the review, ideally using a flow diagram.	Page 9-10<br>Figure 2
	16b	Cite studies that might appear to meet the inclusion criteria, but which were excluded, and explain why they were excluded.	Page 9-10<br>Figure 2
Study characteristics	17	Cite each included study and present its characteristics.	Page 8<br>Table 2
Risk of bias in studies	18	Present assessments of risk of bias for each included study.	Table 2
Results of individual studies	19	For all outcomes, present, for each study: (a) summary statistics for each group (where appropriate) and (b) an effect estimate and its precision (e.g. confidence/credible interval), ideally using structured tables or plots.	Not applicable
Results of syntheses	20a	For each synthesis, briefly summarise the characteristics and risk of bias among contributing studies.	Table 2
	20b	Present results of all statistical syntheses conducted. If meta-analysis was done, present for each the summary estimate and its precision (e.g. confidence/credible interval) and measures of statistical heterogeneity. If comparing groups, describe the direction of the effect.	Not applicable
	20c	Present results of all investigations of possible causes of heterogeneity among study results.	Not applicable
	20d	Present results of all sensitivity analyses conducted to assess the robustness of the synthesized results.	Not applicable
Reporting biases	21	Present assessments of risk of bias due to missing results (arising from reporting biases) for each synthesis assessed.	Not applicable
Certainty of evidence	22	Present assessments of certainty (or confidence) in the body of evidence for each outcome assessed.	Not applicable
**DISCUSSION**			
Discussion	23a	Provide a general interpretation of the results in the context of other evidence.	Page 15-18
	23b	Discuss any limitations of the evidence included in the review.	Page 15-18<br>Table 2
	23c	Discuss any limitations of the review processes used.	Page 18
	23d	Discuss implications of the results for practice, policy, and future research.	Page 17-18
**OTHER INFORMATION**			
Registration and protocol	24a	Provide registration information for the review, including register name and registration number, or state that the review was not registered.	Page 9
	24b	Indicate where the review protocol can be accessed, or state that a protocol was not prepared.	Page 9
	24c	Describe and explain any amendments to information provided at registration or in the protocol.	Not applicable
Support	25	Describe sources of financial or non-financial support for the review, and the role of the funders or sponsors in the review.	Funding Statement
Competing interests	26	Declare any competing interests of review authors.	Funding Statement<br>Page 18
Availability of data, code and other materials	27	Report which of the following are publicly available and where they can be found: template data collection forms; data extracted from included studies; data used for all analyses; analytic code; any other materials used in the review.	Supplementary Table S1

S3 Table: The ENTREQ Checklist

Item	Guide and description	Reported on Page No:
Aim	State the research question the synthesis addresses	7
Synthesis methodology	Identify the synthesis methodology or theoretical framework which underpins the synthesis, and describe the rationale for choice of methodology (e.g. metaethnography, thematic synthesis, critical interpretive synthesis, grounded theory synthesis, realist synthesis, meta-aggregation, meta-study, framework synthesis).	8-9
Approach to searching	Indicate whether the search was pre-planned (comprehensive search strategies to seek all available studies) or iterative (to seek all available concepts until theoretical saturation is achieved).	7
Inclusion criteria	Specify the inclusion/exclusion criteria (e.g. in terms of population, language, year limits, type of publication, study type).	7-8
Data sources	Describe the information sources used (e.g. electronic databases (MEDLINE, EMBASE, CINAHL, psychINFO, Econlit), grey literature databases (digital thesis, policy reports), relevant organisational websites, experts, information specialists, generic web searches (Google Scholar), hand searching, reference lists) and when the searches were conducted; provide the rationale for using the data sources.	7
Electronic search strategy	Describe the literature search (e.g. provide electronic search strategies with population terms, clinical or health topic terms, experiential or social phenomena related terms, filters for qualitative research and search limits).	7-8<br>Supplementary S1 Table
Study screening methods	Describe the process of study screening and sifting (e.g. title, abstract and full text review, number of independent reviewers who screened studies)	8
Study characteristics	Present the characteristics of the included studies (e.g. year of publication, country, population, number of participants, data collection, methodology, analysis, research questions).	9<br>Table 1
Study selection results	Identify the number of studies screened and provide reasons for study exclusion (e.g. for comprehensive searching, provide numbers of studies screened and reasons for exclusion indicated in a figure/flowchart; for iterative searching describe reasons for study exclusion and inclusion based on modifications to the research question and/or contribution to theory development).	9<br>Figure 2
Rationale for appraisal	Describe the rationale and approach used to appraise the included studies or selected findings (e.g. assessment of conduct (validity and robustness), assessment of reporting (transparency), assessment of content and utility of the findings).	8<br>Table 2
Appraisal items	State the tools, frameworks and criteria used to appraise the studies or selected findings (e.g. Existing tools: CASP, QARI, COREQ, Mays and Pope [25]; reviewer developed tools; describe the domains assessed: research team, study design, data analysis and interpretations, reporting).	7-9<br>Table 2
Appraisal process	Indicate whether the appraisal was conducted independently by more than one reviewer and if consensus was required.	8
Appraisal results	Present results of the quality assessment and indicate which articles, if any, were weighted/excluded based on the assessment and give the rationale.	Table 2
Data extraction	Indicate which sections of the primary studies were analysed and how were the data extracted from the primary studies? (e.g. all text under the headings "results /conclusions" were extracted electronically and entered into a computer software).	8-9
Software	State the computer software used, if any	8-9
Number of reviewers	Identify who was involved in coding and analysis.	8-9

Coding	Describe the process for coding of data (e.g. line by line coding to search for concepts).	8
Study comparison	Describe how were comparisons made within and across studies (e.g. subsequent studies were coded into pre-existing concepts, and new concepts were created when deemed necessary).	8-9
Derivation of themes	Explain whether the process of deriving the themes or constructs was inductive or deductive.	8
Quotations	Provide quotations from the primary studies to illustrate themes/constructs, and identify whether the quotations were participant quotations or the author's interpretation	na
Synthesis output	Present rich, compelling and useful results that go beyond a summary of the primary studies (e.g. new interpretation, models of evidence, conceptual models, analytical framework, development of a new theory or construct).	10-15
